# Case Report: Outcome of Osimertinib Treatment in Lung Adenocarcinoma Patients With Acquired KRAS Mutations 

**DOI:** 10.3389/fonc.2021.630256

**Published:** 2021-04-22

**Authors:** Weigang Xiu, Qianqian Zhang, Min Yu, Yin Huang, Meijuan Huang

**Affiliations:** Department of Thoracic Oncology, Cancer Center, West China Hospital, Sichuan University, Chengdu, China

**Keywords:** non–small cell lung cancer, acquired KRAS mutation, osimertinib, immune checkpoint inhibitor, drug-resistance

## Abstract

**Background:**

Osimertinib belongs to the third-generation epidermal growth factor receptor tyrosine kinase inhibitor that has shown positive effects in treating lung adenocarcinoma cancer. However, the subsequent resistance to Osimertinib has become a clinical challenge.

**Case Presentation:**

We present two lung adenocarcinoma cases that developed a resistance to Osimertinib. Among them, one patient attained both KRAS exon 2 and exon 3 mutations and was given paclitaxel (albumin-bound) plus carboplatin. The other patient exhibited a KRAS exon 3 mutation, so the paclitaxel (albumin-bound) plus nivolumab was administered. Eventually, the second patient manifested a better clinical outcome than the first.

**Conclusion:**

These results provide supporting evidence that KRAS exon 3 (R68S) mutations may be associated with Osimertinib resistance in lung adenocarcinoma patients. This further reveals the relationship between subtypes of acquired KRAS mutations and the effect of therapeutic approaches. Moreover, the combination of chemotherapy and immune checkpoint inhibitors may generate a satisfying disease control.

## Introduction

For non–small cell lung cancer (NSCLC) patients harboring epidermal growth factor receptor (EGFR)-activating mutations, the EGFR tyrosine kinase inhibitors (EGFR-TKI) are normally recommended as treatment. As a third-generation EGFR-TKI, Osimertinib is effective in most NSCLC cases. AURA, a phase II clinical trial, had shown that Osimertinib (over response rate [ORR], 71%) was more effective than platinum-based chemotherapy (ORR, 31%) in patients with the EGFR T790M mutation. However, resistance to Osimertinib has gradually emerged in recent years and become an essential challenge in clinical practice as it leads to poor prognosis and has limited treatment options.

KRAS mutations have been considered as one of the underlying mechanisms of Osimertinib resistance (1–5). Typically, 15% to 20% of lung adenocarcinoma patients present with a primary KRAS mutation (6), which occurs mostly in exon 2 and less in exon 3. However, the association between subtypes of acquired KRAS mutations and the effect of therapeutic approaches has yet to be fully established. Here, we presented two such cases and compared the treatment regime and prognosis of the patients.

## Case Presentation

### Case 1

**Figure 1:**
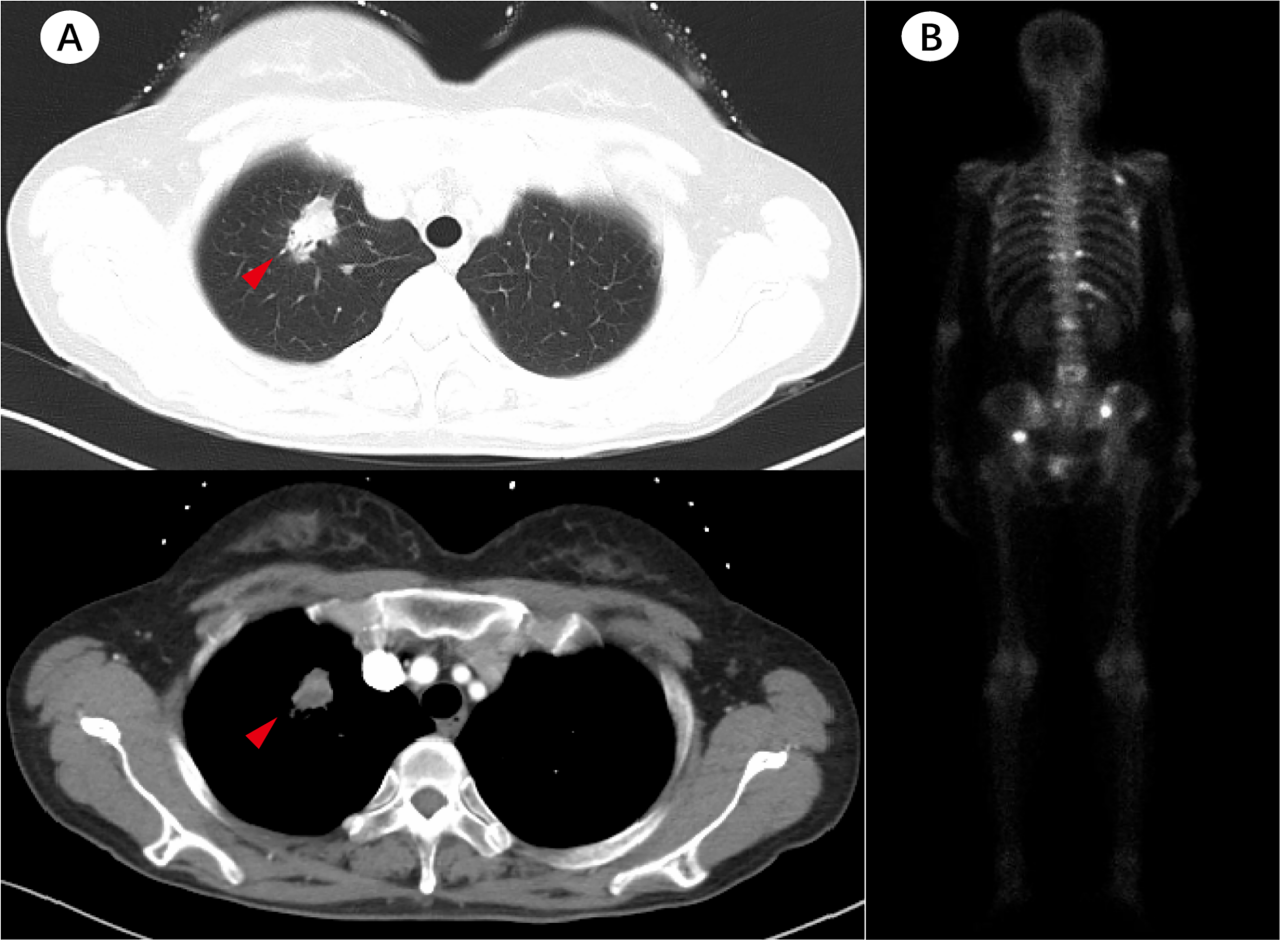
Patient’s imaging before and during anti-tumor treatment. **(A)** The baseline chest computed tomography (CT) scan before treatment. **(B)** Bone isotope scanning suggested extensive bone metastases after 12 months of Osimertinib treatment.

**Figure 2:**
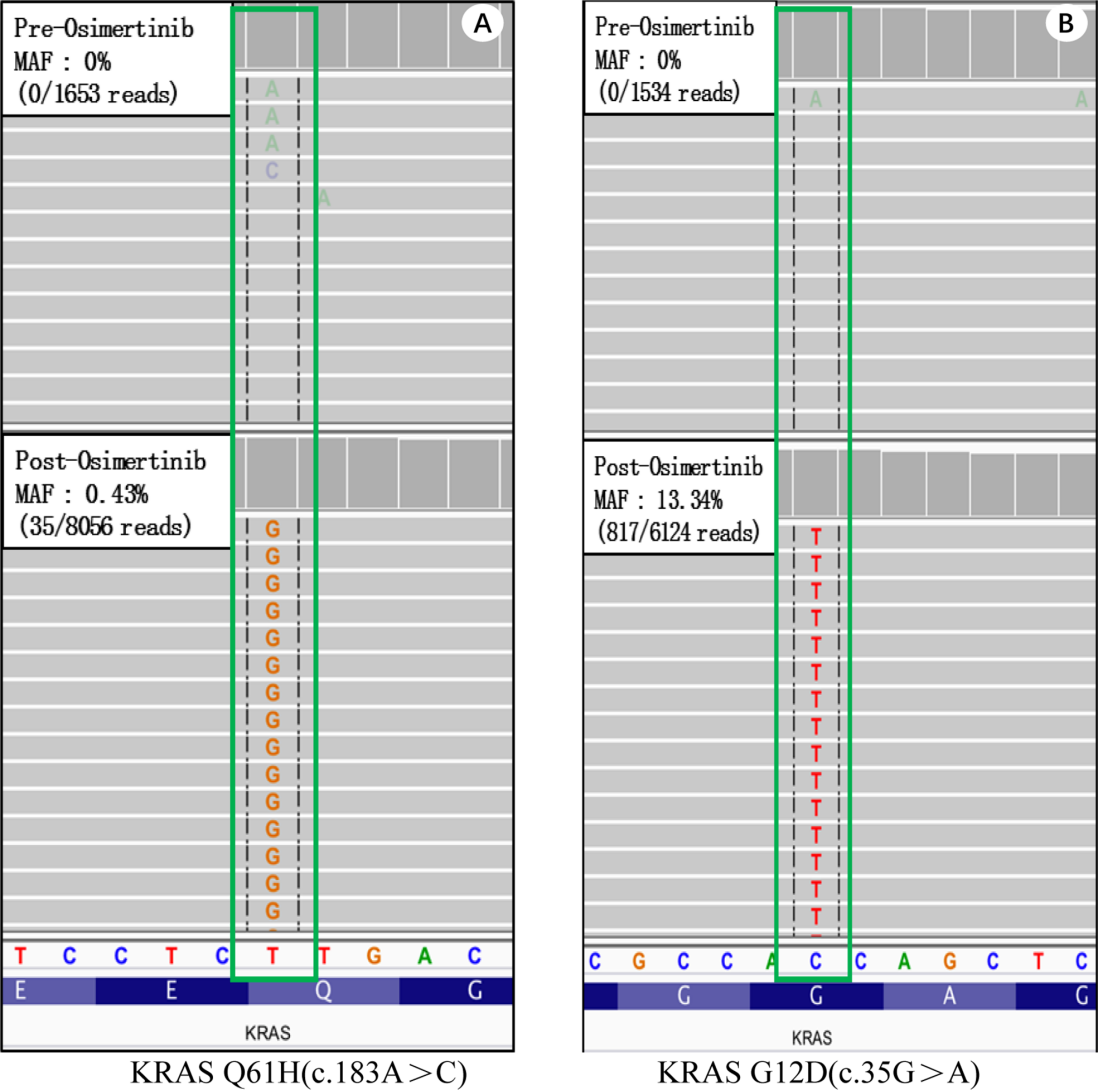
Memorial Sloan Kettering-Integrated Mutation Profiling of Actionable Cancer Targets (MSK-IMPACT) sequencing on tumor tissues of case 1. Wild type KRAS (top) and acquired KRAS mutations. **(A)** Wild type KRAS before treatment (top) and a KRAS Q61H mutation (bottom) after receiving Osimertinib. **(B)** Wild type KRAS before treatment (top) and a KRAS G12D mutation (bottom) after receiving Osimertinib.

A 55-year-old female who has never smoked sought consultation for a cough in October 2016. Her chest computed tomography (CT) showed a lesion in the upper lobe of the left lung (**Figure 1A**) and the patient was diagnosed with lung adenocarcinoma. This patient underwent a right upper lobectomy in November 2016, during which a visceral pleural involvement was detected (pT2aN0M0, stage IB). Postoperative pathology revealed TTF-1 (+), NapsinA (+), TG (−), ALK (−), ROS-1 (−) invasive adenocarcinoma. Afterward, the patient accepted four cycles of adjuvant chemotherapy (pemetrexed plus cisplatin) at a three-month interval. A follow-up contrast-enhanced CT scan showed a spinal metastasis in April 2017 (stage IV). Memorial Sloan Kettering-Integrated Mutation Profiling of Actionable Cancer Targets (MSK-IMPACT) sequencing on the tumor tissue identified an EGFR exon 19 deletion without KRAS mutation. The patient was given gefitinib (250mg/d) and achieved a stable disease state on January 2018 when another contrast-enhanced CT scan detected a spinal metastasis. Molecule genetic testing confirmed EGFR T790M mutation and a negative PD-L1 expression. Then, the patient received Osimertinib (80 mg/d) for 12 months, after which an extensive bone metastasis was found using bone isotope scanning (**Figure 1B**). Re-biopsy of bone metastasis (not decalcification) and another genetic test indicated that the patient was negative for the EGFR-T790 mutation, but positive for the KRAS exon 2 (G12D, MAF:13.34%) and exon 3 (Q61H, MAF:0.43%) mutations, as well as for an EGFR exon 19 deletion (MAF:22.58%) mutation (**Figure 2**). The plasma circulating tumor DNA (ctDNA) confirmed the above results by next Generation Sequencing (NGS). The patient underwent two cycles of paclitaxel (albumin-bound) plus carboplatin for six weeks, which unfortunately did not lead to any apparent benefits. The patient gave up further anti-tumor treatments, including immune checkpoint inhibitors (ICIs), due to his poor physical conditions.

### Case 2

**Figure 3:**
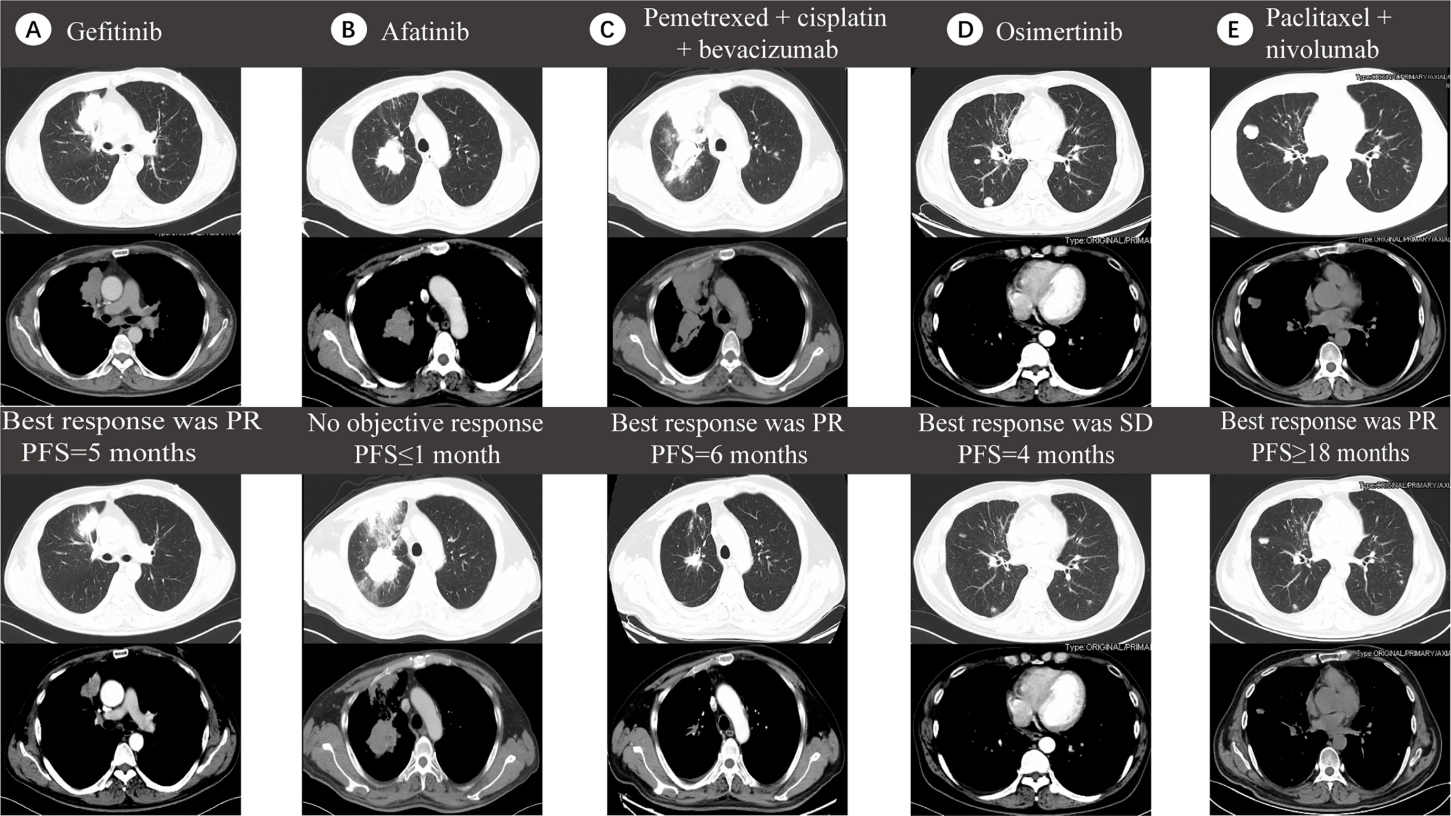
Five lines of treatment for case 2. **(A)** CT scan before (top) and after (bottom) receiving gefitinib, which produced a partial response (PR) in the right lung with 5 months of progression-free survival (PFS). **(B)** CT scan before (top) and after (bottom) receiving afatinib, which showed no objective response with less than one month of PFS. **(C)** CT scan before (top) and after (bottom) receiving pemetrexed, cisplatin and bevacizumab which brought a PR effect with 6 months of PFS. **(D)** CT scan before (top) and after (bottom) receiving Osimertinib, which induced no objective response with four months of PFS. **(E)** CT scan before (top) and after (bottom) receiving paclitaxel (albumin-bound) plus nivolumab which induced a PR effect with more than 18 months of PFS.

In March 2017, a 54-year-old male non-smoker was diagnosed with NSCLC adenocarcinoma metastasizing to double lung, mediastinal and the cervical lymph nodes (cT3N3M1a, stage IV). The pathological biopsy revealed TTF-1 (+), NapsinA (+), CK5/6 (-), TG (−), ALK (−), ROS-1 (−) invasive adenocarcinoma. Next generation sequencing (NGS) on tumor biopsy tissues revealed an EGFR exon 19 deletion without a KRAS mutation. The patient then accepted gefitinib (250 mg/d) which, as shown in contrasted CT, produced a partial response in the right lung (**Figure 3A**). In September 2017, a new lesion in the middle lobe of right lung was found and NGS-based re-biopsy identified both EGFR exon 19 deletion and ERBB3 mutation. Based on these findings, the treatment was changed to afatinib (40 mg/d); however, disease progression in the right lung was soon identified (**Figure 3B**). In December 2017, the patient began receiving a six-cycle chemotherapy regimen with pemetrexed, cisplatin, and bevacizumab, which was partially effective, but new lesions quickly appeared in both lungs (**Figure 3C**). The re-biopsy showed a T790M mutation but tested negative for PD-L1 expression. Osimertinib was administered as the fourth treatment in June 2018. In October 2018, a progression disease (PD) arose in the right lung. Re-biopsy of the right lower lung of the patient revealed no EGFR mutation but tested positive for the KRAS exon 3 (R68S, MAF:5.4%) mutation by MSK-IMPACT sequencing (**Figure 3D**). The plasma ctDNA confirmed the above results by NGS. Therefore, the patient received four cycles of paclitaxel (albumin-bound) plus nivolumab treatment and acquired a partial response (**Figure 3E**).

## Conclusion

The relationship between acquired KRAS mutations and Osimertinib resistance, as well as various therapeutic approaches, have yet to be explored in detail (7, 8). Here we identified two cases where the patients acquired a KRAS mutation after taking Osimertinib and subsequently experienced quick disease progression. These findings are consistent with previous reports. Ortiz et al described a patient who acquired a KRAS G12S mutation with the administration of Osimertinib. Cellular investigation showed that, compared to KRAS wild type transduced cells (PC9^KRAS-wt^), G12S mutation cells (PC9^KRAS G12S^) appeared less sensitive to Osimertinib (9), suggesting that KRAS mutations underlie the resistance to Osimertinib (10–12). Although KRAS and EGFR mutations are thought to exist in a mutually exclusive manner (13), with the introduction of high-sensitivity large-scale mutation analysis, multiple studies have unveiled the co-existence of EGFR mutations and other dominant mutations such as those of KRAS (14). In the current report, both the KRAS mutation and EGFR 19 exon deletion were detected in case 1, suggesting that the KRAS mutation might rescue lung cancer cells from the lethal potency of EGFR TKIs. In addition, it can be inferred that the EGFR TKIs may functionally deplete oncogenic EGFR signal to a degree that would allow co-expression of mutant KRAS and EGFR (15). In addition, the heterogeneity of lung adenocarcinomas may account for these results, and may lead to diversified gene sequencing consequences in different locations of the same primary tumor tissue (16, 17).

In the two cases reported here, the genetic setting and therapeutic outcome showed overt discrepancies. Case 1 exhibited both KRAS exon 2 (G12D) and exon 3 (Q61H) mutation but case 2 only a KRAS exon 3 (R68S) mutation. An R68S mutation is a rare type of KRAS exon 3 mutations (18, 19) and its prognostic role remains controversial (20, 21). A KRAS R68S mutation was reported to be associated with aggressive property of colorectal tumor and acquired resistance to the EGFR inhibitors cetuximab or panitumumab (22). To the best of our knowledge, this is the first study reporting that the KRAS exon 3 (R68S) mutation may be associated with Osimertinib resistance and revealing the relationship between subtypes of acquired KRAS mutations and the effect of therapeutic approaches. It has been previously observed that the KRAS G12D mutation led to the short survival outcome of a never-smoking adenocarcinoma patient (23). This may be due to the change in tumor biological behavior and the sensitivity to treatment that resulted from the differential activation of downstream signaling pathways. Aredo et al also found that KRAS G12D mutations conferred a poor prognosis in NSCLC patients, and moreover acted as a biomarker to predict the benefit of immunotherapy (24). In case 2, when Osimertinib induced disease progression, nivolumab, a type of ICI targeted for PD-L1 was combined with chemotherapy and manifested as a clear favorable consequence. PD-L1 tends to be up-regulated by KRAS mutations through p-ERK signaling which accelerates the apoptosis of CD3-positive T cells. In vitro studies using a co-culture system demonstrated that ICI recovered the anti-tumor immunity of T cells and reduced survival rates of KRAS-mutant NSCLC cells (25). It is on this premise that the immunotherapy may give more flexibility in treating lung cancer with KRAS mutations.

In conclusion, our study provides further evidence supporting the KRAS mutations acting as one of the novel mechanisms for Osimertinib resistance. Moreover, the combined treatment of chemotherapy and ICI exhibits apparent advantages for NSCLC patients with acquired KRAS mutations.

## Data Availability Statement

The original contributions presented in the study are included in the article/supplementary material. Further inquiries can be directed to the corresponding author.

## Ethics Statement

The studies involving human participants were reviewed and approved by The Human Investigation Committee (IRB) of Sichuan University. The patients/participants provided their written informed consent to participate in this study.

## Author Contributions

MH designed the study. YH, QZ and MY conducted the experiments and analyzed the data. WX wrote the manuscript. All authors contributed to the article and approved the submitted version.

## Funding

This work was supported by WU JIEPING MEDICAL FOUNDATION (320.6750.18128).

## Conflict of Interest

The authors declare that the research was conducted in the absence of any commercial or financial relationships that could be construed as a potential conflict of interest.
